# Comparative Study of Vitamin D Levels in Newly Diagnosed Tuberculosis and a Normal Population

**DOI:** 10.3390/medicina60050685

**Published:** 2024-04-23

**Authors:** Vasanth Kumar Mamadapur, Shreesha Nagaraju, Mukhyaprana M. Prabhu

**Affiliations:** Department of General Medicine, Kasturba Medical College, Manipal, Manipal Academy of Higher Education, Manipal 576104, Karnataka, India; vasanth.kumar@manipal.edu

**Keywords:** vitamin D, vitamin D deficiency, tuberculosis, pulmonary tuberculosis, extrapulmonary tuberculosis

## Abstract

*Background and Objectives*: Tuberculosis (TB) is an ancient disease caused by *Mycobacterium tuberculosis*, a member of the *Mycobacterium tuberculosis* complex. It contributes to significant morbidity and mortality. Treatment of TB poses a considerable challenge because of emerging drug resistance and the longer duration of therapy. Various past studies, both in vitro and in vivo, have established the role of vitamin D in the pathogenesis and treatment of TB. Results of in vivo studies are inconsistent, and this study aims to determine vitamin D levels and their association with newly diagnosed TB (pulmonary and extrapulmonary) cases and normal populations. *Material and Methods*: A Prospective Case-Control study with 116 subjects (58 cases and 58 controls) was conducted over two years. 29 cases of pulmonary TB and 29 cases of extrapulmonary TB constituted 58 cases of TB. Vitamin D levels were measured and compared in both the cases and controls. Data analysis was carried out using SPSS software 22.0. *Results*: The prevalence of vitamin D deficiency was 68.96% in the cases, while it was 51.72% in the controls. The reported median and quartile of serum vitamin D levels were 14.35 ng/mL (8.65, 25.48) in the TB group and 19.08 ng/mL (13.92, 26.17) in the control group. There was a significant statistical difference between the TB and non-TB populations with a *p*-value of 0.029 on the Mann–Whitney test. *Conclusion*: Vitamin D deficiency was more prevalent in individuals with TB than those without TB.

## 1. Introduction

Tuberculosis (TB) is an ancient disease. Its cause remained unknown until 24 March 1882, when Dr Robert Koch announced his discovery of the bacillus responsible, subsequently named *Mycobacterium tuberculosis* [1]. TB typically affects the lungs (pulmonary TB) but can also affect other sites—pleura, gastrointestinal tract, kidney, bone, etc. (extrapulmonary TB). Latent TB infection is a persistent immune response to stimulation by *Mycobacterium tuberculosis* (MT) antigens with no evidence of clinically manifest active TB.

TB is a preventable and usually curable disease. Despite this, TB has had a significant impact on global health and was responsible for the second-highest number of deaths from a single infectious agent in 2022, following coronavirus disease (COVID-19), and caused almost twice as many deaths as HIV/AIDS. The reported global number of people newly diagnosed with TB was 7.5 million in 2022. Thirty high-burden countries accounted for 87% of the world’s TB cases in 2022. Two-thirds of the global total was in the following eight countries: India (27%), Indonesia (10%), China (7.1%), Philippines (7.0%), Pakistan (5.7%), Nigeria (4.5%), Bangladesh (3.6%), and the Democratic Republic of the Congo (3.0%). Globally, in 2022, TB caused an estimated 1.30 million deaths [2]. In 2022, 2.42 million TB cases were notified in India [3].

The host susceptibility to TB infection depends on a complex interaction between the host, bacteria, and several factors such as socioeconomic status, malnutrition, overcrowding, immunosuppression, indoor air pollution, alcohol, smoking, etc.

Tuberculosis remains a major public health problem worldwide; it is associated with significant morbidity and mortality. Given the growing number of TB cases, the problem of drug resistance, and the longer duration of therapy, newer treatment modalities must be invented.

Vitamin D (calciferol) and its metabolites are hormones and hormone precursors rather than vitamins since they can be synthesized endogenously in the proper biological setting. Vitamin D from plant sources is vitamin D2 (ergocalciferol), whereas that from animal sources is vitamin D3 (cholecalciferol). The skin is a significant source of vitamin D, which is synthesized upon skin exposure to ultraviolet B radiation (UV-B; wavelength, 290–320 nm).

Vitamin D, whether it is synthesized cutaneously or absorbed from the intestine, is transported through the circulation, bound to vitamin D-binding protein, an α-globulin synthesized in the liver. Vitamin D is subsequently 25-hydroxylated in the liver by a cytochrome P450 oxidase in the mitochondria and microsomes to form 25 hydroxyvitamin D [25(OH)D] (calcidiol). The second hydroxylation, required for the formation of the active hormone 1,25-hydroxyvitamin D [1,25(OH)D] (calcitriol), occurs in the kidney. 25-hydroxyvitamin D [25(OH)D] (calcidiol) is the major circulating and storage form of vitamin D.

The biological effects of vitamin D are mediated by vitamin D receptors, which are found in most tissues. In addition to the classic endocrine effects on calcium and phosphate metabolism and bone health, binding with these receptors potentially expands vitamin D actions to many different cell systems and organs (e.g., immune cells, brain, breast, colon, and prostate).

Vitamin D levels depend on gender, age, diet, season, UV exposure duration, UV rays’ strength, skin pigmentation, and genetics.

The clinical syndrome of vitamin D deficiency can be a result of the deficient production of vitamin D (impaired cutaneous production, lack of dietary intake, malabsorption due to short gut syndrome, gastric bypass surgery), accelerated losses of vitamin D [increased metabolism (barbiturates, phenytoin, rifampin), impaired enterohepatic circulation, nephrotic syndrome, CYP3A4 mutation], impaired vitamin D activation [impaired 25-hydroxylation (liver disease, isoniazid, 25-hydroxylase mutation), impaired 1α hydroxylation—(hypoparathyroidism, ketoconazole, 1α-hydroxylase mutation)], FGF23 (Fibroblast Growth Factor 23) excess—(oncogenic osteomalacia, hypophosphatemic rickets, fibrous dysplasia, chronic kidney disease), target organ resistance (vitamin D receptor mutation, phenytoin), and obesity [4].

Vitamin D deficiency is prevalent globally. Vitamin D deficiency causes Osteomalacia in adults and is associated with various other diseases like Type 2 Diabetes mellitus, Hypertension, Cardiovascular diseases, Cancer, Autoimmune disorders, Influenza, etc. [5]. In their study, Cui, A. et al. found that globally, 15.7%, 47.9%, and 76·6% of participants had serum 25-hydroxyvitamin D levels less than 12, 20, and 30 ng/mL, respectively [6]. Siddiqee, M.H. et al., in their study in South Asian countries, concluded that the highest prevalence of vitamin D deficiency was found in Pakistan (73%), followed by Bangladesh (67%), India (67%), Nepal (57%), and Sri Lanka (48%) [7].

Around two centuries ago, Chapman reported the efficacy of administering cod liver oil, which is rich in vitamin D, in patients afflicted with TB. This approach was found to yield improved clinical outcomes [8]. In 1859, Germany established the first sanatorium to treat TB patients. Patients were exposed to fresh air at high altitudes, given nutritious food, and advised to rest [9]. Exposing TB patients to sunlight, also known as heliotherapy, gained significant popularity and became a prevalent method for enhancing vitamin D synthesis in their skin [10]. Niels Ryberg Finsen demonstrated that short-wave UV light was effective against cutaneous TB and won the Nobel Prize for Medicine in 1903 [11]. Vitamin D is essential in preventing infection of MT through various mechanisms.

Various studies have been performed in the past that correlate vitamin D levels and TB. Most of them studied only pulmonary TB. The results of various previous studies are inconsistent; few studies have assessed vitamin D levels in pulmonary and extrapulmonary TB. Hence, this study compared the vitamin D levels in TB (pulmonary and extrapulmonary) and healthy controls.

## 2. Materials and Methods

### 2.1. Study Design

A Prospective Case-Control study with 116 subjects (58 cases and 58 controls) was conducted at Kasturba Hospital, a Tertiary Care Centre in Manipal, Udupi, India, over two years. Subjects were recruited after fulfilling the Inclusion and Exclusion criteria. Institutional Ethical Committee approval was obtained, and written informed consent was taken from each study participant.

Inclusion criteria:

Patients with age >18 years.

Patients diagnosed with TB with AFB (Acid Fast Bacilli) or GeneXpert-positive.

Exclusion criteria:

Patients with chronic liver disease and chronic kidney disease.

Patients receiving vitamin D supplementation.

Patients with HIV-positive status.

Pregnant patients.

### 2.2. Data Collection

A Case proforma was used to collect the patient details such as age, gender, socioeconomic status, clinical history, and physical examination findings. Subjects were recruited into two groups based on the presence or absence of TB as cases and controls, respectively. Microbiologically proven (AFB or GeneXpert) TB was taken as a case (pulmonary TB—29 and extrapulmonary TB—29). Rifampicin resistance was not seen in any of the cases. Patients without symptoms of TB and whose vitamin D laboratory values were available were taken as controls. Serum calcium, serum phosphorus, serum albumin, and ESR values were measured. Our laboratory measured vitamin D levels using an Electrochemiluminescence-automated analyser (ECLIA) blood test.

Vitamin D status was classified as vitamin D deficiency <20 ng/dL, vitamin D insufficiency 21–30 ng/dL, and vitamin D sufficiency >30 ng/dL based on the clinical practice guidelines of the Endocrine Society Task Force on Vitamin D [12]. Vitamin D levels were compared in both the groups of cases and controls. Additionally, vitamin D levels in extrapulmonary TB patients were analysed.

### 2.3. Statistical Analysis

Data analysis was performed using SPSS software 22.0. Results were presented as mean with SD for normally distributed data and as median with interquartile range (IQR) for skewed data. Continuous variables were analysed using the Mann–Whitney U test. Associations between the categorical variables were analysed using Fisher’s exact or chi-square tests. The statistical significance was set at *p*-value ≤ 0.05.

**Figure 1:**
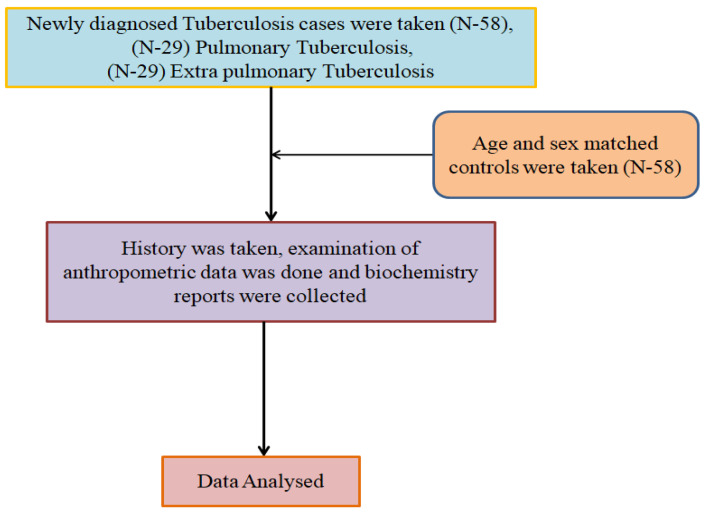
Data collection.

The Summary of data collection is represented in Figure 1 as shown below.

## 3. Results

### 3.1. Characteristics of the Study Population

After applying the specified selection criteria, 116 study participants (58 cases and 58 controls) were chosen for this study. Among these subjects were 63 males (54.31%) and 53 females (45.68%), with a mean age of 49.95 ± 14 years. The study population’s characteristics, including age (years), gender, albumin (mg/dL), body mass index (BMI), haemoglobin (g/dL), corrected calcium (mg/dL), ESR (mm/h), platelets (Lakhs/dL), total count (10^3^/L), and comorbidities like hypertension and diabetes are listed in Table 1.

The cases group exhibited lower mean levels of albumin (3.15 ± 0.66 mg/dL) and corrected calcium (9.09 ± 0.59 mg/dL) compared with the controls, where albumin was (4.51 ± 0.24), and corrected calcium was (9.46 ± 0.41), and the difference was found to be statistically significant.

ESR levels were much higher in the cases than in the controls. The mean and SD of ESR was 45 ± 24.89 mm/h for the cases and 10.18 ± 8 mm/h for the control group.

The frequency of chronic diseases like Diabetes and Hypertension was similar between the case and control groups, with 37.9% of the cases and 36.2% of the controls being individuals with Diabetes. Both groups displayed identical distributions of these conditions.

### 3.2. Characterization of Pulmonary TB and Extrapulmonary TB

The cases were categorized into pulmonary TB and extrapulmonary TB groups following the guidelines of the RNTCP (Revised National Tuberculosis Control Programme). Table 2 represents a summary of participant characteristics for both types of TB, including gender, age, albumin levels (mg/dL), body mass index (BMI), ESR (mm/h), corrected calcium levels (mg/dL), and the presence of comorbidities such as Hypertension and Diabetes mellitus.

### 3.3. Distribution of Different Types of Extrapulmonary TB in the Cases

**Figure 2:**
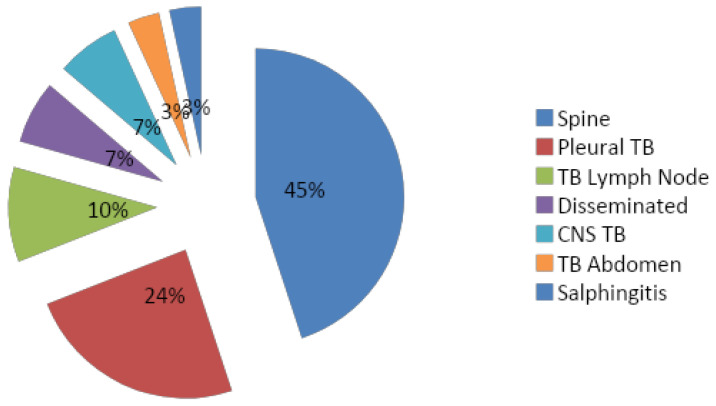
Pie chart representing different types and frequencies of extrapulmonary TB.

The pie chart in Figure 2 displays the occurrence of different categories of extrapulmonary TB cases within our selected study population. The most prevalent cases were observed in TB spine—13 (44.83%), followed by pleural TB—7 (24.14%), TB lymph node—3 (10.34%), disseminated TB—2 (6.89%), CNS TB—2 (6.89%), TB abdomen—1 (3.45%), and TB salpingitis—1 (3.45%).

### 3.4. Prevalence of Vitamin D Deficiency among Both the Cases and the Controls

**Figure 3:**
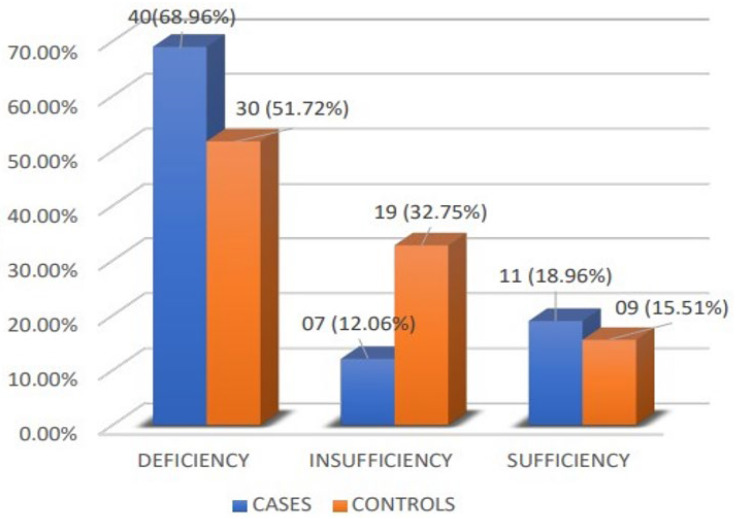
Bar chart comparing different categories of vitamin D in the cases and controls.

The prevalence of vitamin D deficiency was 68.96% (40) in the cases and 51.72% (30) in the controls. Vitamin D insufficiency was observed in 12.06% (07) of the cases, and sufficiency was seen in 18.96% (11), as shown in Figure 3. There was a statistically significant difference between the cases and control groups with a *p*-value of 0.028 by the chi-square test.

### 3.5. Prevalence of Vitamin D Deficiency in Pulmonary and Extrapulmonary TB

**Figure 4:**
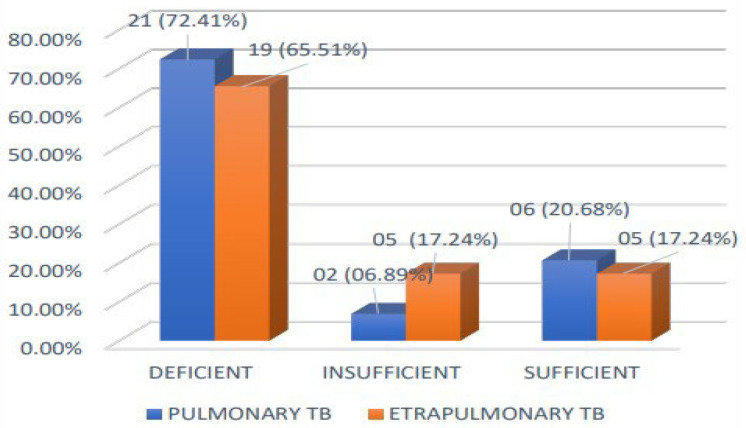
Bar chart comparing different categories of vitamin D in pulmonary and extrapulmonary TB.

The prevalence of Vitamin D deficiency was observed in 72.4% (21) of the subjects in the pulmonary TB group and 65.51% (19) of the subjects in the extrapulmonary TB group. Among the pulmonary TB cases, 6.89% (2) were vitamin D insufficient. In comparison, 17.24% (5) of the extrapulmonary TB cases were deficient in vitamin D. The data on the percentage of pulmonary and extrapulmonary TB cases with vitamin D deficiency, insufficiency, and sufficiency are represented in the bar graph Figure 4.

### 3.6. Vitamin D Levels in the Cases and Controls

**Figure 5:**
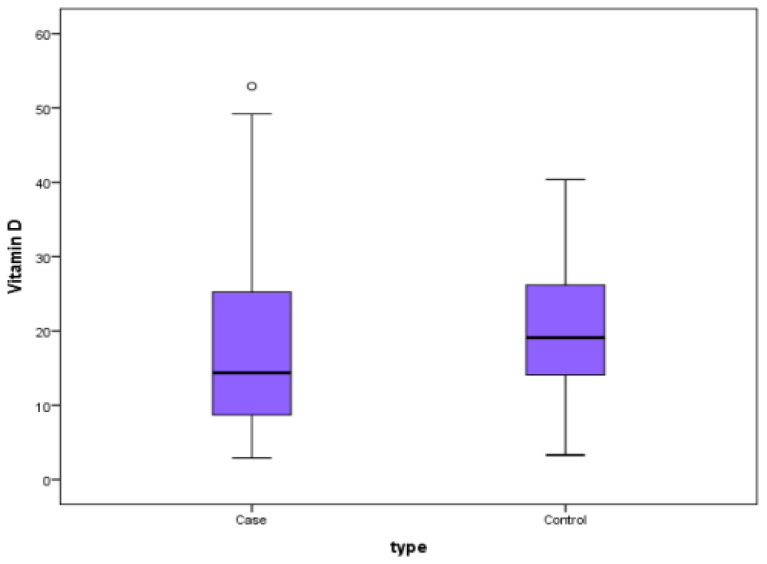
Box–whisker plot comparing average vitamin D levels in the cases and controls.

The reported median and quartile of serum vitamin D levels were 14.35 ng/mL (8.65, 25.48) in the tuberculosis group and 19.08 ng/mL (13.92, 26.17) in the control group. There was a significant statistical difference between the TB cases and non-TB populations with a *p*-value of 0.029 on the Mann–Whitney test. The box–whisker plot in Figure 5 summarizes the results of the serum vitamin D levels (ng/dL) in the cases and controls.

### 3.7. Vitamin D Levels in Pulmonary and Extrapulmonary TB

**Figure 6:**
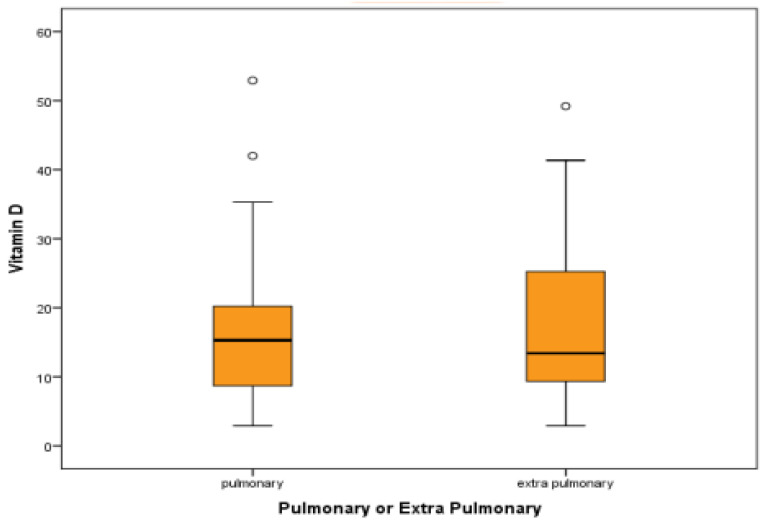
Box–whisker plot comparing average vitamin D levels in pulmonary TB and extrapulmonary TB.

The reported median and quartile of serum vitamin D levels were 15.30 (8.63, 23.47) in the TB group and 13.40 (8.47, 25.72) in the extrapulmonary TB group. Though the serum vitamin D levels were lower in the extrapulmonary TB group than in pulmonary TB, there was no significant statistical difference between the groups. A *p*-value of 0.9 was found using the Mann–Whitney test. The box–whisker plot in Figure 6 summarizes the results of the serum vitamin D levels (ng/dL) in the pulmonary and extrapulmonary TB cases.

### 3.8. AFB Grading and Pulmonary TB

**Figure 7:**
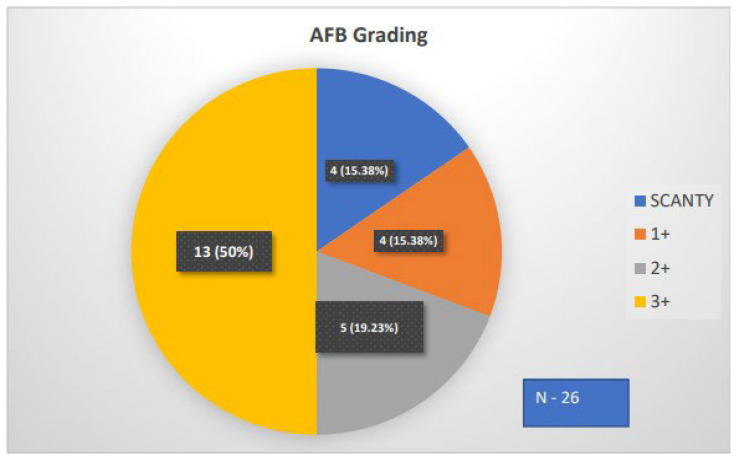
Pie chart representing the frequency of AFB categories.

Of the 29 pulmonary TB cases, 26 cases were positive for sputum AFB. Grading was based on bacterial load. Half of the cases (50%) were in 3+, and the remaining were equally distributed. The pie chart in Figure 7 summarizes the AFB grading and pulmonary TB results.

### 3.9. Correlation of Sputum Bacterial Load with Vitamin D Levels

#### 3.9.1. AFB Grading and Vitamin D Levels

**Figure 8:**
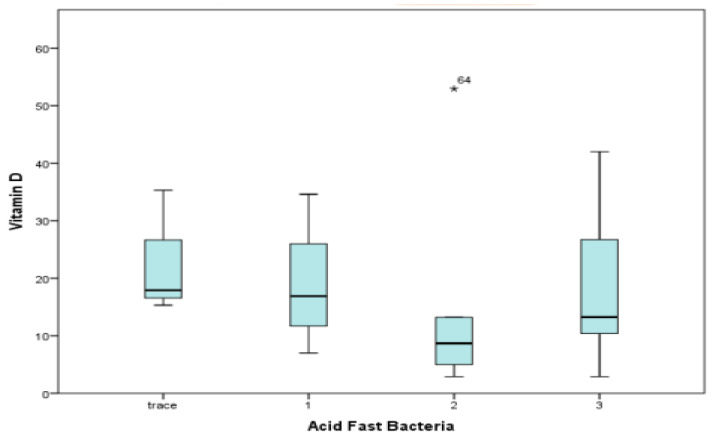
Correlation of sputum AFB grading and vitamin D levels.

Among the pulmonary TB cases, 26 were AFB-positive and were grouped based on AFB grading. No statistical correlation was found between different grading of sputum AFB positivity and the median vitamin D levels among groups using the Pearson chi-square test. The box–whisker plot in Figure 8 summarizes the results of the bacterial load correlation with vitamin D levels, where the Pearson chi-square test returned a *p*-value of 0.4.

#### 3.9.2. GeneXpert Categories and Vitamin D Levels

**Figure 9:**
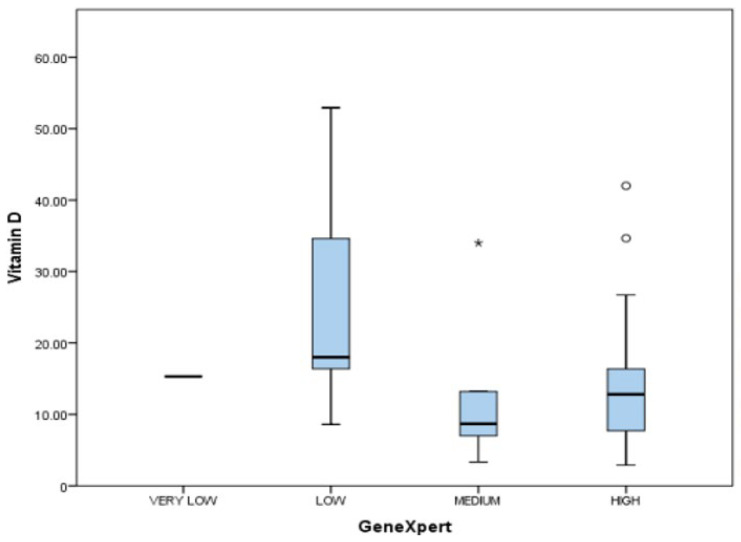
Correlation between GeneXpert categories and vitamin D levels.

Among the 29 pulmonary TB cases, 28 were positive for GeneXpert and were grouped based on GeneXpert categories as per the bacterial load. The box–whisker plot in Figure 9 summarizes the results of the bacterial load correlation with vitamin D levels. No statistical correlation was found between mean vitamin D levels among different GeneXpert categories and the Pearson chi-square test. Pearson chi-square test = *p*-value of 0.301.

## 4. Discussion

This study included 116 subjects with 58 cases and 58 age- and sex-matched controls after fulfilling the Inclusion and Exclusion criteria. Of the 58 cases, 29 patients had pulmonary TB and 29 had extrapulmonary TB.

Most studies performed to evaluate vitamin D levels in TB included only pulmonary TB. Very few studies in the recent past have included both pulmonary TB and extrapulmonary TB [13,14,15,16]. Hammami, F. et al., in their study, included only patients with extrapulmonary TB [17], and Pareek et al. predominantly studied vitamin D deficiency in extrapulmonary TB [18].

In the present study, among the 29 extrapulmonary TB patients, the most prevalent cases were observed in TB of spine—13 (44.83%) followed by pleural TB—7 (24.14%), TB lymph node—3 (10.34%), disseminated TB—2 (6.89%), CNS TB—2 (6.89%), TB abdomen —1 (3.45%), and TB salpingitis—1 (3.45%). In a few studies, TB pleural effusion was the predominant site of extrapulmonary TB [13,14,16,19]. In other studies, the TB lymph node was the predominant site of extrapulmonary TB [15,17,18].

### 4.1. Prevalence of Vitamin D Deficiency in the Cases and Controls

Vitamin D deficiency was seen in 68.95% of the cases and 51.72% of the controls. Like our study, other studies showed vitamin D deficiency more in cases than controls [19,20,21,22]. In a few studies, vitamin D deficiency was seen only in cases but none in controls [13,14,23,24].

### 4.2. Mean Value of Vitamin D in the Cases and Controls

The recorded quartile and median serum vitamin D values were 14.36 ng/mL (8.63, 25.42) in the TB group and 19.07 ng/mL (13.82, 26.27) in the control group. The application of the Mann–Whitney test indicated a significant statistical difference (*p*-value of 0.029) between the TB cases and the non-TB populations. Like our study, other studies also revealed low vitamin D levels in the TB group compared with the control group [13,14,20,22,23,24].

### 4.3. Prevalence of Vitamin D Deficiency in Pulmonary TB and Extrapulmonary TB

Vitamin D deficiency was observed in 72.4% (21) of individuals in the pulmonary TB group and 65.51% (19) of subjects in the extrapulmonary TB groups. Like our study, Sariata et al. found that vitamin D deficiency was more prevalent in pulmonary TB [13]. In Soleimani, Alireza et al. and Sethiya B et al.’s studies, vitamin D deficiency was more prevalent in extrapulmonary TB than pulmonary TB [15,23].

### 4.4. Mean Value of Vitamin D in Pulmonary TB and Extrapulmonary TB

The quartile and median values of vitamin D levels were reported as 15.32 (8.64, 23.43) in the pulmonary TB group and 13.42 (8.47, 25.72) in the extrapulmonary TB group. Although no significant statistical difference was recorded between the groups, it was noteworthy that the extrapulmonary TB group showed lower levels of vitamin D compared with the pulmonary TB group.

In their studies, Soleimani, Alireza, et al. and Pareek et al. showed a lower mean value of vitamin D in extrapulmonary TB compared with pulmonary TB [15,18]. Contrary to our study, Sariata et al. and Balgi, V. et al. revealed that patients with extrapulmonary TB had a higher mean vitamin D level than patients with pulmonary TB [13,14].

Kafle, S. et al., in their meta-analysis, revealed low levels of vitamin D in patients with pulmonary TB compared with healthy people [25]. Several other studies in different populations have reported an association between vitamin D deficiency and increased risk of pulmonary tuberculosis [26,27,28,29].

Cai, L. et al., Ralph et al., and Friis et al. are among the few studies concluding that patients with TB were likely to have a higher vitamin D level than healthy participants [30,31,32].

### 4.5. Vitamin D Levels and AFB Grading

Our study showed no significant statistical correlation between Vitamin D levels and sputum AFB grading. In contrast to our study, various studies have revealed that low vitamin D levels were associated with higher bacillary load [33,34,35,36].

Vitamin D plays a vital role in MT infection through different mechanisms.

MT enters macrophages through toll-like receptors (TLRs) on their surface. Once inside, the TLRs activate a signalling pathway, exposing the macrophages to inflammatory cytokines. This exposure increases the expression of CYP27B1 oxidase, which oxidizes 25(OH)D to the active form 1,25(OH)2D. 1,25(OH)2D activates the signalling pathway mediated by the VDR/RXR receptors on the macrophages through an autocrine mechanism, which leads to the synthesis of the Leucine–Leucine-37 peptide (LL-37). This peptide is derived from cathelicidin hCAP-18 and destroys bacterial cells by interacting with bacterial cell wall molecules and perforating the cytoplasmic membrane [37,38,39,40].

Studies have shown that vitamin D can induce autophagy of infected macrophages [41,42,43]. Miley, A. et al., in their study, showed that co-administration of vitamin D and phenylbutyrate led to a significant increase in LL-37 levels in macrophages and lymphocytes, resulting in heightened intracellular killing of MT [44]. Vitamin D has been found to effectively hinder the growth of MT in infected macrophages by producing nitrogen and oxygen reactants [45]. Vitamin D is known to stimulate the production of methyl glycol and β-Fenin 2 (an antimicrobial peptide), which helps attract monocytes, neutrophils, and T cells to the site of infection. This plays a significant immunomodulatory role in the treatment of tuberculosis [46]. As per some studies, vitamin D deficiency has been linked to the absence of some anti-mycobacterial activities mediated by T-helper lymphocytes [47,48]. The TACO (Tryptophan aspirate-containing Coat protein) gene plays a vital role in MT survival. Vitamin D, along with Retinoic acid, has been shown to down-regulate gene transcription in macrophages, consequently inhibiting the survival of MT [49].

Vitamin D has an anti-inflammatory activity that limits an excessive inflammatory response, causing tissue damage through various mechanisms [50,51,52].

Polymorphism of vitamin D receptors and vitamin D binding protein can influence TB susceptibility and response to anti-tubercular drug treatment [53,54].

The human vitamin D receptor (VDR) is a nuclear hormone receptor encoded by the VDR gene on chromosome 12q. The VDR gene is a polymorphic gene, and various SNPs (single nucleotide polymorphisms) have been reported. Fok (rs2228570), Taq (rs731236), Bsm (rs1544410), and Apa1 (rs7975232) are the most studied SNPs in TB patients. Several studies conducted globally on different populations have found a variation in the response to both the disease and therapy for TB [53].

Srishthi Shah et al., in their trial sequential meta-analysis, found that genetic variations in the Fok1 and Bsm1 VDR genes provided protection against the development of TB. The gene polymorphism in Apa1 was associated with the development and progression of TB. However, the genetic variations in Taq1 seemed to play no role [55]. Upendra Yadav et al. discovered a significant association between FokI and TB susceptibility in the overall analysis and the Asian population [56]. Sudhasini Panda et al. demonstrated a considerable association between the Fok1 SNP (f allele) and susceptibility to TB [57].

In their study, Dauren Yerezhepov et al. found no statistically significant correlation between any of the four VDR polymorphisms (FokI, TaqI, ApaI, and BsmI) and TB in the co-dominant model, which equally assessed the contribution of each genotype. However, they did find a statistically significant association between the BsmI (rs1544410) polymorphism of the VDR gene and TB in the recessive model [58].

In their meta-analysis, Bin Li et al. found a significant correlation between the VDR polymorphism of the TaqI gene and susceptibility to TB in Iranians and Indians. However, no association was found between the vitamin D receptor polymorphism TaqI and the Chinese population [59]. In their study, Mukhtar Sadykov et al. found a significant association between VDR SNPs rs1544410, BsmI and rs731236, TaqI and TB [60]. Asadollah Mohammadi et al., in their study, demonstrated the significant effect of the TaqI polymorphism in all different genetic models and the dominant genotype of the BsmI polymorphism on increased risk of TB in an Iranian population. However, FokI and ApaI did not show any significant effects on the development of TB [61].

Vitamin D binding protein (VDP) is a multifunctional protein highly expressed and encoded on chromosome 4. Once vitamin D and its metabolites are circulated, they bind with VDP. The most extensively studied gene in the vitamin D metabolic pathway is the VDP gene. The two most studied SNPs in the VDB gene are rs4588 and rs7466 [53].

Murugesan Harishankar et al., in their study, suggest that the rs4588 “CA” genotype is significantly associated with susceptibility to TB, while the rs4588 “AA” genotype is associated with protection against TB [62].

Tian-Ping Zhang et al., in their study, provided evidence that the GC rs3733359, rs16847024, and rs4588 variants might contribute to pulmonary TB susceptibility in the Chinese population [63].

A few studies have reported the association between vitamin D and Latent Tuberculosis Infection (LTBI), and they produced inconsistent and varying results. According to a study by Cao, Y. et al., there appears to be no significant association between serum vitamin D levels and the incidence of LTBI. Moreover, their study findings suggest that relatively high serum vitamin D levels are unlikely to be a protective factor for LTBI [64]. B Patterson et al. showed an independent association between vitamin D deficiency and progression from LTBI to active disease [65]. Arnedo-Pena et al., in their study, showed that LTBI was not associated with vitamin D status, but severe vitamin D deficiency was associated with increased TB infection conversion [66].

The anti-mycobacterial properties of vitamin D in vitro are well established, and several mechanisms by which it exerts this action have been identified. However, the role of vitamin D in preventing and treating TB, as observed in in vivo studies, has yielded inconsistent and conflicting results.

A few studies have shown that supplementing vitamin D can prevent latent TB or active TB [67], while others have not shown any benefit in preventing TB [68,69].

According to a study conducted by Harish Chandra et al., adjunctive vitamin D3 may have a potential role in expediting the resolution of inflammatory responses and enhancing clinical outcomes for patients diagnosed with pulmonary TB [70]. Wen, Y. et al., in their study, found that in patients with TB and 25(OH)D deficiency, calcitriol supplementation could elevate CD4+ T cell levels, shorten the time to sputum culture conversion, and accelerate lesion absorption [71]. Karbalaei, M. et al. suggested that vitamin D could be recommended for adjunctive therapy for TB, in combination with anti-tuberculosis drugs, and for prophylactic aims [72]. In their study, Hong-Xia Wu et al. concluded that vitamin D supplementation could be considered a combination therapy in patients with pulmonary TB [73].

Other studies inferred that vitamin D administered with standard treatment had no beneficial effect on TB patients compared with a placebo [74,75].

Variability in the results of different studies could be due to the lack of a standard definition of vitamin D deficiency, the type of vitamin D measured (calcidiol/calcitriol), and different types and doses of vitamin D administered to study participants, polymorphisms of the genes that code for vitamin D receptors and vitamin D binding protein, interaction of vitamin D with anti-tubercular drugs, other vitamins and micronutrients deficiencies, and environmental and social factors.

A few of the limitations of our study were the uneven distribution of participants among various groups, which affected the vitamin D and AFB correlation. Consideration was not given to diet, duration of sunlight exposure, or skin pigmentation.

## 5. Conclusions

Vitamin D deficiency was more prevalent in patients with TB than in the healthy controls. There was no statistically significant difference in the prevalence of vitamin D deficiency in pulmonary and extrapulmonary TB. Among the pulmonary TB population, our study did not find a statistically significant correlation between vitamin D levels and sputum bacterial load.

Even though many conditions affect vitamin D levels in the body and low vitamin D levels are not specific to TB, a strong association is present between low levels of vitamin D and TB. Vitamin D is inexpensive, readily available, and has a low incidence of side effects, even at relatively higher dosages. Further research is required to determine its efficacy in vivo, remove confounding factors, and specify the optimal dosages, duration, type, and administration methods for the prevention and treatment of TB.

## Figures and Tables

**Table 1:** Characteristics of the study population in the cases and controls.

	Cases (*n* = 58) Mean ± SD	Control (*n* = 58) Mean ± SD	Total (116) Mean ± SD	*p*-Value
Age (years)	50 ± 16.46	49 ± 11.38	49.95 ± 14	
Gender				
Male	31 (53.4%)	32 (55.2%)	63 (54.31%)	
Female	27 (46.6%)	26 (44.8%)	53 (45.68%)	
BMI (kg/m^2^)	20.69 ± 4.47	25.60 ± 4	23.15 ± 4.89	0.00
Albumin (mg/dL)	3.15 ± 0.66	4.51 ± 0.24	3.83 ± 0.84	0.00
Corrected calcium (mg/dL)	9.09 ± 0.59 (*n* = 51)	9.46 ± 0.41 (*n* = 57)	9.29 ± 0.54	0.00
ESR (mm/h)	45 ± 24.89	10.18 ± 8	28.06 ± 25	0.00
Haemoglobin (g/dL)	11.13 ± 1.82	13.43 ± 2.08	12.28 ± 2.26	
Total count (10^3^/L)	9831 ± 4018	6658 ± 1593	8244 ± 3434	
Platelets (Lakhs/dL)	3.87 ± 1.37	2.63 ± 0.81	3.25 ± 1.28	
Comorbidities				
Diabetes	22 (37.9%)	21 (36.2%)	43 (37.06%)	
Hypertension	8 (13.79%)	15 (25.86%)	23 (19.82%)	

Body mass index (BMI); erythrocyte sedimentation rate (ESR).

**Table 2:** Characteristics of the study population based on the site of the infection.

	Pulmonary (*n* = 29)	Extrapulmonary (*n* = 29)	*p*-Value (*t*-Test)
Age (years)	52.62 ± 14.1	48.27 ± 18.44	
Gender			
Male	16 (55.2%)	15 (51.7%)	
Female	13 (44.8%)	14 (48.3%)	
BMI (kg/m^2^)	19.82 ± 4.24	21.57 ± 4.59	0.13
Corrected calcium (mg/dL)	9.08 ± 0.67	9.1 ± 0.52	0.55
ESR (mm/h)	51.51 ± 22.78	40.57 ± 26.05	0.1
Comorbidities			
Diabetes	16 (55.17%)	6 (20.68%)	
Hypertension	5 (17.24%)	3 (10.29%)	

Body mass index (BMI); erythrocyte sedimentation rate (ESR).

## Data Availability

Data will be shared by the corresponding author upon request.
